# Interglial Crosstalk in Obesity-Induced Hypothalamic Inflammation

**DOI:** 10.3389/fnins.2018.00939

**Published:** 2018-12-13

**Authors:** Md Habibur Rahman, Min-Seon Kim, In-Kyu Lee, Rina Yu, Kyoungho Suk

**Affiliations:** ^1^Department of Pharmacology, Brain Science and Engineering Institute, BK21 Plus KNU Biomedical Convergence Program, School of Medicine, Kyungpook National University, Daegu, South Korea; ^2^Division of Endocrinology and Metabolism, University of Ulsan College of Medicine, Seoul, South Korea; ^3^Department of Internal Medicine, Division of Endocrinology and Metabolism, School of Medicine, Kyungpook National University, Daegu, South Korea; ^4^Department of Food Science and Nutrition, University of Ulsan, Ulsan, South Korea

**Keywords:** obesity, diabetes, hypothalamus, inflammation, glia, chemokine, metabolism

## Abstract

Glial cells have recently gained particular attention for their close involvement in neuroinflammation and metabolic disorders including obesity and diabetes. In the central nervous system (CNS), different types of resident glial cells have been documented to express several signaling molecules and related receptors, and their crosstalks have been implicated in physiology and pathology of the CNS. Emerging evidence illustrates that malfunctioning glia and their products are an important component of hypothalamic inflammation. Recent studies have suggested that glia–glia crosstalk is a pivotal mechanism of overnutrition-induced chronic hypothalamic inflammation, which might be intrinsically associated with obesity/diabetes and their pathological consequences. This review covers the recent advances in the molecular aspects of interglial crosstalk in hypothalamic inflammation, proposing a central role of such a crosstalk in the development of obesity, diabetes, and related complications. Finally, we discuss the possibilities and challenges of targeting glial cells and their crosstalk for a better understanding of hypothalamic inflammation and related metabolic dysfunctions.

## Introduction

The hypothalamus is a critical brain structure and consists of three major areas such as the periventricular, the medial, and the lateral hypothalamic area. The median eminence (ME) is located in the middle–basal hypothalamus and the dorsal side of the third ventricle, and has blood vessels without barrier, which act as a window to release hypothalamic metabolic signals (Peruzzo et al., 2000; Elizondo-Vega et al., 2015). Nuclei of each area are metabolically important and play crucial roles in diverse functions including nutrient sensing, appetite control, and energy metabolism (Coll and Yeo, 2013; Elizondo-Vega et al., 2015). The hypothalamus receives metabolic signals from periphery through hormonal and cellular communication, letting the hypothalamus to control whole-body metabolism (Alquier and Kahn, 2004). However, disturbance of this mechanism is a major concern in a broad range of metabolic diseases including obesity and diabetes. Rising evidence suggests that hypothalamic inflammation particularly in the arcuate nucleus (ARC), a part of periventricular area, is a common pathological feature related with obesity and diabetes (Valdearcos et al., 2015). It affects the endocrine system and disturbs nutritional homeostasis, which causes metabolic alterations and subsequent pathologies. In animal experiments, high-fat diet (HFD)-induced chronic inflammation in the hypothalamus has been documented to modulate feeding behavior, energy expenditure, insulin secretion, and hepatic glucose production, all of which have been associated with dysregulation of glucose and fatty acid (FA) metabolism in obesity and diabetes (Cai and Liu, 2011). It has been reported that hypothalamic inflammation is observed within first 3 days of HFD onset with increased level of inflammatory markers (Thaler et al., 2012; Baufeld et al., 2016). An animal study of neonatal overnutrition revealed that hypothalamic inflammation is age- and sex-dependent, which is observed only in male animals with significant weight gain at postnatal day 150 (Argente-Arizon et al., 2018). The possible reason of such differences is the elevated level of circulating estrogens in female animals, probably inhibiting the effects of FAs on inflammation. As in other brain regions, the hypothalamus also contains various types of non-neuronal cells such as microglia, astrocytes, tanycytes, and NG2-positive glial cells (oligodendrocyte progenitor cells), which all play important physiological roles as metabolic sensors in the hypothalamus (Garcia-Caceres et al., 2012; Elizondo-Vega et al., 2015; Djogo et al., 2016; Freire-Regatillo et al., 2017). Emerging evidence suggests that glial cells may be a key component of pathological hypothalamic inflammation as well. Notably, wide structural changes have been observed in hypothalamic astrocytes in animal models of obesity and type 2 diabetes (Horvath et al., 2010). This study suggests that astrocytes contribute to synaptic rewiring by altering the glial ensheathment of neurons, which may disturb the ability of neurons to receive direct nutritional signals from the periphery. In line with this thought, it has been proposed that hypothalamic inflammation is a potential cause of leptin and insulin resistance in the hypothalamus of rodents (Thaler et al., 2012; Andre et al., 2017) and patients with obesity and diabetes (Schur et al., 2015; Kreutzer et al., 2017).

In recent years, glial cells have become a focus of modern neuroscience research, for instance, with respect to neurodevelopment and brain health/disease (Barres, 2008). They are homeostatic regulators in the central nervous system (CNS) and play a critical role by providing metabolic support for neurons. In addition, they maintain extracellular pH, protect neurons from any insults, as well as participate in neurovascular coupling, neurotransmitter uptake, synaptic formation and pruning, and phagocytosis (Jha and Suk, 2013; Jha et al., 2014). Moreover, glial cells participate in these functions by communicating with and influencing other glial cell types in a mutual manner, ultimately playing a crucial role in brain development, function, and diseases (Jha et al., 2018). This interglial communication occurs through various cell surface molecules and the release of signaling molecules such as cytokines, chemokines, adenosine triphosphate, and growth factors (Tanuma et al., 2006; Pascual et al., 2012; Rocha et al., 2012; Liddelow et al., 2017). It has been reported that glial crosstalk plays a crucial role in the development and progression of neuropathologies depending on the level of their secreted molecules following various CNS insults (Tian et al., 2012). Among different glial cell types, microglia and astrocytes are the primary innate immune cells and key responders to a variety of CNS insults such as pathogens, ischemia, and chemogenic factors, which further lead to the activation of these glial cells and ensue the production of increased levels of proinflammatory mediators, for example, cytokines, chemokines, reactive oxygen species, and nitric oxide (NO) (Tanuma et al., 2006; Iizumi et al., 2016; Hou et al., 2017; Liddelow et al., 2017). These mediators are responsible for chronic inflammation and resultant pathologies in the CNS. However, several molecular targets such as anti-inflammatory cytokines and neurotrophic factors have been identified in other brain areas where the inflammatory response due to microglia and astrocytic activation has been reduced in Alzheimer’s disease (Mandrekar-Colucci et al., 2012), Parkinson disease (PD) (Subramaniam and Federoff, 2017), and epilepsy (Benson et al., 2015). It has been reported that activated microglia-derived IL-10 reduces inflammatory responses in the stratum, which has been proposed as a crucial target to enhance the neuroprotection in an experimental model of PD (Schwenkgrub et al., 2013; Joniec-Maciejak et al., 2014).

Emerging evidence highlights that microglia, astrocytes, and their interaction play an important role in the regulation of immune and inflammatory responses in the CNS (Farina et al., 2007; Ransohoff and Brown, 2012; Kirkley et al., 2017). In the context of metabolic inflammation in the hypothalamus, the interaction between these glial cell types has been reported to be a decisive contributor to progression and severity of neurometabolic diseases (Kim et al., 2018). In addition, glial interaction modulates their activation states, particularly through the expression of cell surface receptors including cytokine and chemokine receptors, and activation of intracellular signaling pathways (Zhang et al., 2017; Kim et al., 2018; Valdearcos et al., 2018), potentially amplifying the inflammatory activation in the hypothalamus under diabetes and obesity conditions (Rahman et al., 2018). A recent study has revealed that inflammatory activation of astrocytes by lipid droplets and the potential crosstalk of these astrocytes with microglia in free FA (FFA)-rich environments might be implicated in the obesity-induced pathological hypothalamic inflammation and subsequent metabolic complications (Kwon et al., 2017).

**FIGURE 1:**
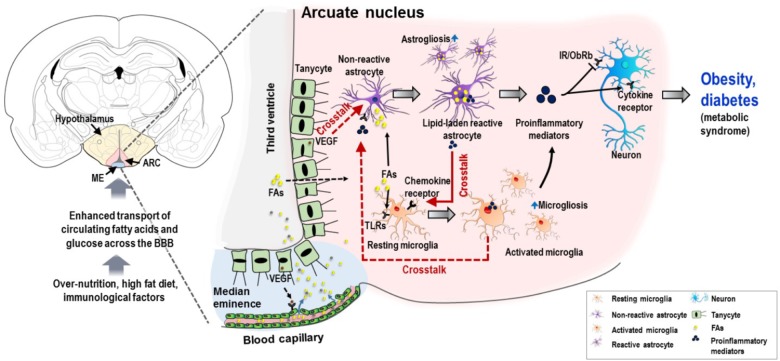
Schematic representation of potential interglial crosstalk in hypothalamic inflammation and the ontology of obesity and diabetes. Overnutrition, HFD, immunological factors, and increased circulating nutrients (FAs and glucose), arising in a temporal sequence, appear to induce glial malfunctions and pathological hypothalamic inflammation. The excessive circulating nutrients are transported into the hypothalamus through the permeable blood capillaries. Tanycytes, a specialized ependymal cell type located in the lateral walls and surface of the third ventricle, allow the passage of these excessive nutrients particularly FAs from the circulation to the hypothalamic ARC. This may be due to the expression of VEGF in tanycytes, which targets microvessel loops and promotes microvessel permeability. FAs can activate both microglia and astrocytes, primarily by binding to TLRs expressed in microglia. Similarly, FAs cause accumulation of lipid droplets in astrocytes, leading to inflammatory activation of astrocytes and release of proinflammatory mediators, which may further activate microglia by molecular interaction enhancing the inflammatory processes. In addition, activated microglia and VEGF-rich tanycytes may also contribute to chronic hypothalamic inflammation by their secreted molecules and subsequent crosstalk with astrocytes. This proinflammatory mechanism impairs insulin and leptin signaling in neurons, leading to cellular stress responses. This condition negatively affects the proper functioning of hypothalamic neuronal circuits, which leads to metabolic dysregulation associated with obesity and diabetes. Solid red arrows, reported glial crosstalk; dotted red arrows, possible crosstalk. BBB, blood–brain barrier; ARC, arcuate nucleus; FA, fatty acid; TLR, toll-like receptor; VEGF, vascular endothelial growth factor; IR, insulin receptor; ObRb, leptin receptor b.

This review discusses the recent advances in the molecular aspects of interglial crosstalk in hypothalamic inflammation, particularly astrocyte–microglia crosstalk, with extended discussion on the potential crosstalks between microglia–astrocyte and tanycyte–astrocyte, suggesting a central role of the interglial crosstalk in the development of obesity, diabetes, and their complications, based on the data currently available in the literatures (Figure 1). Finally, we also discuss the possibilities and challenges of targeting glial cells and their crosstalk for a better understanding of hypothalamic inflammation and its related metabolic dysfunctions.

## Chemokine-Mediated Astrocyte–Microglia Crosstalk

Chemokines are a superfamily of structurally related small (most of them between 8 and 14 kDa) chemotactic cytokines, i.e., they promote leukocyte migration, and are implicated in various inflammatory pathological processes and infectious diseases. In the healthy brain, different cell types including glial cells or neurons constitutively express many chemokines and their receptors, and their expression levels are found to be increased in activated astrocytes and microglia (Le Thuc et al., 2017). Among those, some chemokines such as monocyte chemoattractant protein-1 (MCP-1/CCL2), stromal cell-derived factor (SDF-1/CXCL12), and fractalkine/CX3CL1 and their receptors have been shown to be expressed in the hypothalamus (Banisadr et al., 2005), and their association with obesity-induced inflammatory responses and metabolic complications have been reported (Morari et al., 2014; Poon et al., 2016).

**FIGURE 2:**
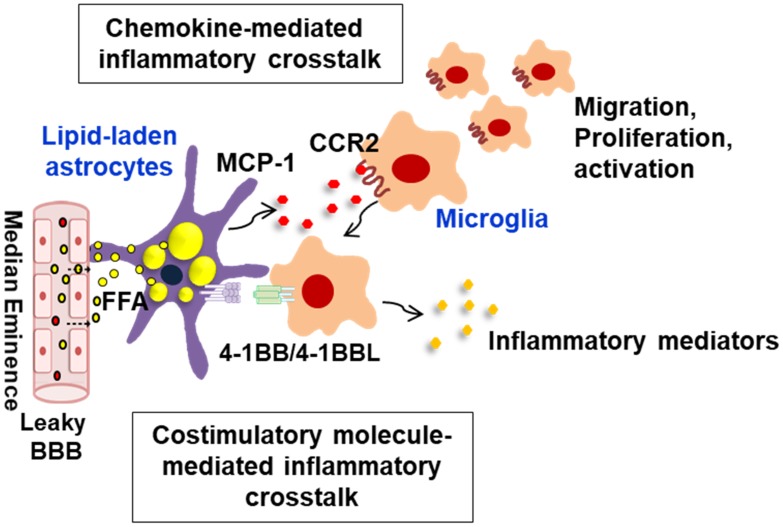
Schematic representation of interglial crosstalk via chemokines and costimulatory molecules in hypothalamic inflammation under obese conditions. In the hypothalamus, glial cells such as astrocytes and microglia can directly respond to peripheral excess nutrient signals, such as FAs, through a unique vascular system (fenestrated capillaries) and damaged BBB and these glial cells become reactive to release inflammatory mediators. In obese environments rich in FAs, astrocytes accumulate lipid droplets, and they release the proinflammatory chemokine MCP-1, which promotes microglia migration, proliferation, and activation. The lipid-laden astrocytes can also directly interact with the migrated microglia through membrane-bound costimulatory receptors/ligands such as 4-1BB/4-1BBL, thus reinforcing the inflammation. This glial cell-mediated inflammatory crosstalk may be crucial for obesity-induced hypothalamic inflammation. BBB, blood–brain barrier; FFA, free fatty acid; MCP-1, monocyte chemoattractant protein-1; CCR2, C–C chemokine receptor type 2.

The chemokine MCP-1/CCL2 is expressed in glial cells such as astrocytes and microglia, and central delivery of MCP-1 has been shown to promote hypothalamic inflammation and metabolic dysregulation as well (Le Thuc et al., 2016). Intriguingly, a recent study has demonstrated that astrocyte-derived MCP-1 is crucial for the onset and/or progress of the obesity-induced glial cell-mediated hypothalamic inflammation (Kwon et al., 2017). *In vitro* experiment using primary hypothalamic astrocytes has revealed that the cells accumulate lipid droplets under FFA-rich conditions with their increased inflammatory reactivity, mimicking obese conditions. The lipid-laden astrocytes produce large amounts of proinflammatory mediators such as tumor necrosis factor (TNF)-α, interleukin (IL)-1β, IL-6, and MCP-1, suggesting that under obese conditions these lipid-laden astrocytes may trigger the onset of hypothalamic inflammation. Of note, treatment of microglia with fatty astrocytes-conditioned medium markedly increased the chemotactic activity of microglia with upregulation of CCR2 (receptor for MCP-1/CCL2), and treatment of anti-MCP-1 antibody diminished the chemotactic activity. These results indicate that the fatty astrocytes-derived inflammatory chemokine MCP-1 enhances microglial chemotactic activity by binding to its receptor, and the recruited microglia may interact with the neighboring astrocytes, amplifying the hypothalamic inflammatory responses (Figure 2). It has been reported that mice fed with HFD show the increased number of microglia in ARC region of the hypothalamus. The study suggested that the increased number of microglia in the ARC might be due to increased infiltration of bone marrow-derived monocytes/macrophages into the CNS (Thaler et al., 2012; Buckman et al., 2014; Valdearcos et al., 2014). Given that the chemokine MCP-1 disrupts the integrity of the blood–brain barrier (BBB) (Yao and Tsirka, 2014), the chemokine MCP-1 may also act in obesity as a signal to recruit bone marrow-derived monocytes/macrophages into the hypothalamus.

In recent years, chemokines such as SDF-1/CXCL12 and fractalkine/CX3CL1 have been shown to be associated with obesity-induced hypothalamic inflammation. SDF-1 is constitutively expressed in hypothalamic astrocytes, microglia, as well as in neurons (Banisadr et al., 2003). SDF-1 and its receptors CXCR4 and CXCR7 have been shown to be upregulated in the hypothalamic nuclei by HFD intake, which is accompanied by an increase of orexigenic neuropeptides (Poon et al., 2016). Moreover, central administration of CXCL12 increases caloric intake, stimulates the expression of orexigenic neuropeptides, and reduces novelty-induced locomotor activity, suggesting that SDF-1 might have an important role in mediating the HFD-induced neuronal and behavioral effects. The crosstalk between astrocytic SDF-1 and microglial CXCR4 has been shown to participate in the development of neuroinflammation and the pathogenesis of neuropathic pain hypersensitivity (Luo et al., 2016). Fractalkine/CX3CL1 is predominantly expressed and released by neurons, and its receptor CX3CR1 is expressed in microglia (Tian et al., 2012). Fractalkine and its receptor play a pivotal role in the recruitment, infiltration, and proinflammatory polarization of leukocytes and microglial cells that are involved in the regulation of neuroinflammatory conditions (Cardona et al., 2006; Cardona et al., 2008), indicating that the chemokine acts as a mediator in the crosstalk between leukocytes and microglia. Fractalkine has been shown to exert both anti-inflammatory (Zujovic et al., 2000) and proinflammatory properties (Huang et al., 2006; Denes et al., 2008), however, recent studies support the proinflammatory role of fractalkine in obesity-induced hypothalamic inflammation. In obesity-prone mice, the inhibition of hypothalamic fractalkine reduces obesity-related inflammatory activity as demonstrated by the reduction of TNF-α and IL-1β levels and the decreased recruitment of bone marrow-derived cells to the hypothalamus. This model also prevents the development of obesity and glucose intolerance (Morari et al., 2014), indicating that fractalkine is a key player in diet-induced early hypothalamic inflammation. Chemokines such as SDF-1 and fractalkine are considered interesting therapeutic targets to fight against obesity-induced inflammatory complications; however, detailed molecular mechanisms underlying the chemokine-mediated interglial crosstalk in obesity-induced hypothalamic inflammation remain to be further investigated.

## Interglial Crosstalk Mediated by Costimulatory Molecules

The contribution of glia–glia interaction to hypothalamic inflammation is strongly influenced by their potential for mutual interaction with the inflammatory microenvironment. Substantial evidence points to reactive glia as a pivotal factor during the obesity-induced hypothalamic inflammation process. As mentioned earlier, astrocytes and microglia in the hypothalamus are often found in close proximity to each other, indicating a potential reciprocal contact-dependent communication between them, facilitating their inflammatory crosstalk.

Several immune signaling costimulatory molecules (ligands/their receptors, membrane-bound proteins), which are mostly expressed in immune cells, glial cells, and neurons, and their crosstalk has been implicated in CNS inflammatory pathologies. For instance, different TNF receptor superfamily (TNFRSF) members and their ligands such as TWEAK/FN14 and CD40/CD40L contribute to the pathogenesis of Alzheimer’s disease and multiple sclerosis, which have a strong inflammatory component and glia involvement (Aarts et al., 2017). CD40/CD40L interaction has been reported to enhance the inflammatory response in multiple sclerosis, and genetic ablation or antibody-mediated inhibition of the CD40/CD40L interaction reduces the severity of experimental autoimmune encephalomyelitis (EAE), a murine model for multiple sclerosis (Gerritse et al., 1996; Grewal et al., 1996; Aarts et al., 2017). The 4-1BB (also known as TNFRSF9 or CD137) receptor/ligand system may be another potent mediator in controlling neuroinflammatory pathologies. It has been reported that mice with 4-1BB ligand (4-1BBL) deficiency display profoundly less microglial activation following EAE induction compared with wild-type animals, and, thus, the gene deficiency reduces the severity of this neuroinflammatory pathology (Yeo et al., 2012). These costimulatory receptors/ligands are, therefore, considered to be attractive therapeutic candidates for diverse neuroinflammatory diseases. However, studies on the association of these receptors/ligands system with obesity-induced hypothalamic inflammation are currently limited.

Nonetheless, there is recent evidence supporting the occurrence of interglial crosstalk through 4-1BB/4-1BBL interaction in the hypothalamus under obese conditions (Kim et al., 2018). 4-1BB is a member of the TNFRSF and a membrane-bound costimulatory protein, which is highly expressed in activated T cells as well as NK cells (Vinay and Kwon, 2011). It has also been reported to be expressed by some nonimmune cells such as adipocytes and endothelial cells (Drenkard et al., 2007; Kim et al., 2011) and delivers potent costimulatory or inflammatory signals upon activation. 4-1BB binds to its ligand 4-1BBL, which is highly expressed in antigen-presenting cells such as macrophages. In addition, the 4-1BB receptor/ligand system can transmit bidirectional signals between two interacting cells, a process referred to as forward and reverse signaling (Shao and Schwarz, 2011). Although most studies are focused on 4-1BB/4-1BBL-mediated immune regulations, a few recent studies have shown that the 4-1BB/4-1BBL-mediated bidirectional signal can promote obesity-induced adipose tissue inflammation by the recruitment of adipocytes/macrophages and inflammation in skeletal muscle by myotubes/macrophages recruitment (Tu et al., 2012; Le et al., 2013). Intriguingly, astrocytes express 4-1BB, whereas microglia express 4-1BBL (Reali et al., 2003; Yeo et al., 2012), and these molecules are likely to have the potential to mediate the crosstalk between astrocytes and microglia. Indeed, it has been shown that mice fed with HFD display an increased expression of 4-1BB and 4-1BBL transcripts in the hypothalamus when compared with lean controls (Kim et al., 2018), supporting the idea that these molecules may be responsible for the crosstalk between astrocytes and microglia in obese conditions. Using mouse primary astrocytes and BV-2 mouse microglial cells *in vitro*, the involvement of these molecules in glial cells-mediated hypothalamic inflammatory responses was assessed in a recent study (Kim et al., 2018). First, obesity-related factors such as lipopolysaccharide, FFA, glucose, and adipose tissue-conditioned medium treatment increased the transcript levels of 4-1BB in astrocytes and 4-1BBL in microglia, mimicking the conditions of obesity. Second, stimulation of 4-1BB on astrocytes delivered an inflammatory signal to increase astrocyte reactivity, leading to an increased production of proinflammatory mediators such as TNF-α, IL-6, and MCP-1 at the levels of mRNA and protein. Third, 4-BBL stimulation of microglial cells increased microglial activation, enhanced the release of proinflammatory mediators such as MCP-1 and ICL-6, and decreased the level of an anti-inflammatory cytokine IL-10. This was mediated through activation of the mitogen-activated protein kinase pathway. Fourth, an increased production of inflammatory cytokines was observed in the co-culture of glial cells with augmented glial activation markers. More importantly, inhibition of 4-1BB/4-1BBL crosstalk with a neutralizing antibody decreased the inflammatory responses in the co-cultured glial cells. Moreover, 4-1BB-deficient astrocytes revealed a reduction in the inflammatory responses when co-cultured with microglia, compared with those astrocytes/microglia isolated from wild-type animals. Finally, consistent with these findings, an *in vivo* study revealed that 4-1BB deficiency reduces HFD-induced hypothalamic inflammation, which is accompanied by the reduction of glial activation markers and the level of inflammatory cytokines. Taken all together, these findings suggest that 4-1BB and/or 4-1BBL signaling plays a crucial role in the interglial crosstalk in hypothalamic inflammatory responses under obese conditions (Figure 2) and might be a potential therapeutic target for the inhibition of obesity-induced hypothalamic inflammation and associated metabolic complications.

## Tanycytes and Their Potential Crosstalk

Tanycytes are specialized ependymal cells. Anatomically, this cell type is located in the lateral walls and surface of the third ventricle. Tanycytes maintain the nutrient transport from the circulation to the energy-sensing ARC of the hypothalamus (Mullier et al., 2010; Rahman et al., 2018). According to their location and morphological distribution, tanycytes are classified as two major subtypes (α- and β-tanycytes), which are further subdivided into α1-, α2-, β1-, and β2-tanycytes (Rizzoti and Lovell-Badge, 2017). Specifically, α-tanycytes reside dorsally, while β-tanycytes occupy the ventral side wall and surface of the third ventricle in the ME. Previous studies revealed that HFD increases the proliferation of β2-tanycytes with postnatal neurogenesis in the ME. It is also found that the new born neurons are derived from the β2-tanycytes, and inhibition of tanycyte division and differentiation decreases the HFD-induced body weight gain (Lee and Blackshaw, 2012; Lee et al., 2012). These findings suggest that β2-tanycytes-derived neurogenesis might contribute to energy homeostasis in response to HFD. In the hypothalamus, tanycytes are a source of vascular endothelial growth factor (VEGF) (Langlet, 2014). It has been reported that in mice under food deprivation condition, tanycytes express VEGF-A, which targets microvessel loops to promote microvessel permeability and to reorganize tight junction complexes in the ME and ARC (Langlet et al., 2013). This, in turn, may facilitate the communication between circulating metabolites and brain parenchyma. In a previous study, microglia-secreted VEGF has been shown to regulate the pathogenic activities of astrocytes in an EAE mouse model of multiple sclerosis (Rothhammer et al., 2018). In line with this thought, tanycytes-derived VEGF can be a potential contributor to overnutrition-induced hypothalamic inflammation by interacting with astrocytes. It has been reported that mice fed a HFD show an early loss of the structural organization of β1-tanycytes (Ramalho et al., 2018) in the ME. In this study, immunofluorescence analyses revealed that consumption of an HFD alters the expression and spatial distribution of different proteins including insulin-like growth factor-binding protein 2, the most specific marker of β1-tanycytes, involved in the organization of the BBB. This may facilitate the transport of excess lipid-rich nutrients into the hypothalamus. A previous animal study revealed that hypothalamic tanycytes also accumulate lipid droplets in normal condition (Brion et al., 1982). A recent study using a microscopy analysis of the lipid droplet distribution in the hypothalamus has demonstrated numerous lipid droplets in tanycytes in response to HFD or obese condition (Hofmann et al., 2017). Further research is required to better understand the acute and chronic effects of HFD on tanycyte characteristics in energy metabolism and the role of tanycyte interaction with other glial cells in hypothalamic inflammation and subsequent pathologies.

## Conclusion and Future Perspectives

Accumulating evidence suggests that hypothalamic glial cells respond to diet rich in FAs. In particular, long-chain FAs, such as palmitic acid, have been implicated in microglial activation and subsequent initiation of intracellular inflammatory pathways through toll-like receptors (Milanski et al., 2009; Valdearcos et al., 2014). It has been reported that mice fed with HFD show an activation and proliferation of both microglia and astrocytes in the hypothalamus, and activation of these glial cells play a crucial role in the alteration of local neuronal circuits, which has been suggested to promote obesity (Horvath et al., 2010; Andre et al., 2017). An *in vitro* study using primary microglia and astrocytes isolated from mice brains have revealed that only microglia, but not astrocytes, respond to FAs (Valdearcos et al., 2014). Indeed, FA-mediated inflammatory activation of primary microglia cultures isolated from whole mouse brains or of BV-2 mouse microglial cells is associated with an increased release of proinflammatory mediators, such as TNF-α, IL-1β, and IL-6 (Wang et al., 2012). These findings suggest that HFD-induced inflammatory activation of astrocytes in the hypothalamus might be due to microglial crosstalk with astrocytes through interaction between microglia-secreted proinflammatory mediators and related receptors expressed in astrocytes. In contrast, a previous study reported that FAs, such as palmitic acid, stearic acid, and lauric acid, can activate inflammatory signaling leading to the release of cytokines in cultured astrocytes isolated from rat brain (Gupta et al., 2012). These previous findings suggest that HFD-induced hypothalamic inflammation might be mediated by both glial cell types through their direct or indirect molecular interaction. The contrast in the data in the literature might be due to differences in experimental conditions or species. Further studies are required to explain these discrepancies.

Several *in vivo* and *in vitro* studies provided in the last few years a new view on the roles of microglia–astrocyte interaction involved in the pathogenesis of neuroinflammatory and degenerative diseases (Jha et al., 2018). In addition, bidirectional communication between microglia and astrocytes has been reported to mediate prolonged inflammation via their released molecules such as TNF-α, NO, nicotinamide adenine dinucleotide phosphate (NADPH) oxidase-derived hydrogen peroxide (H_2_O_2_), and transforming growth factors following CNS insults (Jha et al., 2018). Similarly, in the context of metabolic inflammation in the hypothalamus, several animal studies have revealed an increased level of such molecules in the hypothalamus and their role in pathological hypothalamic inflammation associated with obesity and diabetes (Duparc et al., 2011; Dalvi et al., 2017; Katashima et al., 2017; Mendes et al., 2018), but the molecular mechanisms underlying the interglial crosstalk in relation to chronic inflammation in the hypothalamus need to be further highlighted in future research. Glial cells and their inflammatory activation in response to fat-rich diet are closely involved in chronic hypothalamic inflammation and leptin/insulin resistance, which have drawn growing attention to the role of these glial cells in metabolic dysregulation associated with obesity and diabetes. Various secreted molecules from malfunctioning glia and signaling from cell surface receptors have been proposed as a potential mechanism of hypothalamic inflammation. Recent studies have provided a significant insight into our understanding of the complex roles of glial cells and their crosstalk in overnutrition-induced chronic hypothalamic inflammation and its pathological consequences. The complex heterogeneous nature of each glial cell type has made a challenging task to gain a clear insight into the interglial crosstalk in CNS physiology as well as pathologies. Further investigations will be needed for a better understanding regarding the functional significance of the interglial crosstalk in such pathological hypothalamic inflammation. Recently established innovative tools, such as isolation technique of high-purity glial cell populations from rodents and humans, optogenetics, chemogenetics, nanotechnology, single-cell multi-omics, induced pluripotent stem cells, and organoids may accelerate preclinical research.

Diverse glia-secreted molecules including cytokines, chemokines, and gliotransmitters, as well as related receptors expressed in specific glial cell types, can be possible targets for a further clarification of the interglial crosstalk in chronic hypothalamic inflammation. A recent study has demonstrated that restoring the feeding pattern by blocking gap-junction hemichannels in the CNS prevents HFD-induced obesity in mice (Sasaki et al., 2018). It has been suggested that blockade of hemichannels inhibits microglial release of small molecules, without attenuating acute inflammatory cytokine induction. In line with these findings, the authors speculated that HFD-induced obesity can be mediated via the gap junction hemichannel pathway, which affects the timing of meal and the inflammatory cytokine signaling pathway, thereby revealing a new therapeutic target in the CNS for the treatment of obesity. As gap-junction hemichannels control the release of diverse microglial molecules, it is important to understand their association with interglial crosstalk in both acute and chronic phase of obesity. Current and future research may boost the feasibility of glia-based therapeutic strategies targeting the interglial crosstalk and related molecules (either secreted or cell surface molecules) to prevent and alleviate hypothalamic inflammation and metabolic diseases.

## Author Contributions

All authors have made a substantial intellectual contribution to this work, and approved submission of the manuscript. RY and KS formulated the focus of this review. MR, M-SK, and I-KL conducted the literature review and participated in the discussion. MR, RY, and KS wrote the manuscript.

## Conflict of Interest Statement

The authors declare that the research was conducted in the absence of any commercial or financial relationships that could be construed as a potential conflict of interest.
