# Corrigendum: Interglial Crosstalk in Obesity-Induced Hypothalamic Inflammation

**DOI:** 10.3389/fnins.2019.00003

**Published:** 2019-01-23

**Authors:** Md Habibur Rahman, Min-Seon Kim, In-Kyu Lee, Rina Yu, Kyoungho Suk

**Affiliations:** ^1^Department of Pharmacology, Brain Science and Engineering Institute, BK21 Plus KNU Biomedical Convergence Program, School of Medicine, Kyungpook National University, Daegu, South Korea; ^2^Division of Endocrinology and Metabolism, University of Ulsan College of Medicine, Seoul, South Korea; ^3^Department of Internal Medicine, Division of Endocrinology and Metabolism, School of Medicine, Kyungpook National University, Daegu, South Korea; ^4^Department of Food Science and Nutrition, University of Ulsan, Ulsan, South Korea

**Keywords:** obesity, diabetes, hypothalamus, inflammation, glia, chemokine, metabolism

There is an error in the funding statement. The correct funding statement is as follows: “KS was supported by a grant from the Korea Healthcare Technology R&D Project, Ministry of Health and Welfare, South Korea (HI16C1501) and the Basic Science Research Program through the National Research Foundation (NRF), which is funded by the Korean Government (MSIP) (2018R1A2A1A05077118, 2016M3C7A1904148, and NRF-2017R1A5A2015391). M-SK was supported by a grant from the National Research Foundation of Korea funded by the Korean Government (2017R1A2B3007123).”

The authors apologize for this error and state that this does not change the scientific conclusions of the article in any way. The original article has been updated.

## Conflict of Interest Statement

The authors declare that the research was conducted in the absence of any commercial or financial relationships that could be construed as a potential conflict of interest.

